# Natural Time Analysis: The Area under the Receiver Operating Characteristic Curve of the Order Parameter Fluctuations Minima Preceding Major Earthquakes

**DOI:** 10.3390/e22050583

**Published:** 2020-05-21

**Authors:** Nicholas V. Sarlis, Efthimios S. Skordas, Stavros-Richard G. Christopoulos, Panayiotis A. Varotsos

**Affiliations:** 1 Section of Condensed Matter Physics, Department of Physics, National and Kapodistrian University of Athens, Panepistimiopolis, 157 84 Zografos, Greece; nsarlis@phys.uoa.gr (N.V.S.); eskordas@phys.uoa.gr (E.S.S.); 2 Solid Earth Physics Institute, Department of Physics, National and Kapodistrian University of Athens, Panepistimiopolis, 157 84 Zografos, Greece; ac0966@coventry.ac.uk; 3 Faculty of Engineering, Environment and Computing, Coventry University, Priory Street, Coventry CV1 5FB, UK

**Keywords:** natural time analysis, order parameter of seismicity, entropy change under time reversal, entropy, ROC

## Abstract

It has been reported that major earthquakes are preceded by Seismic Electric Signals (SES). Observations show that in the natural time analysis of an earthquake (EQ) catalog, an SES activity starts when the fluctuations of the order parameter of seismicity exhibit a minimum. Fifteen distinct minima—observed simultaneously at two different natural time scales and deeper than a certain threshold—are found on analyzing the seismicity of Japan from 1 January 1984 to 11 March 2011 (the time of the M9 Tohoku EQ occurrence) 1 to 3 months before large EQs. Six (out of 15) of these minima preceded all shallow EQs of magnitude 7.6 or larger, while nine are followed by smaller EQs. The latter false positives can be excluded by a proper procedure (*J. Geophys. Res. Space Physics* 2014, 119, 9192–9206) that considers aspects of EQ networks based on similar activity patterns. These results are studied here by means of the receiver operating characteristics (ROC) technique by focusing on the area under the ROC curve (AUC). If this area, which is currently considered an effective way to summarize the overall diagnostic accuracy of a test, has the value 1, it corresponds to a perfectly accurate test. Here, we find that the AUC is around 0.95 which is evaluated as outstanding.

## 1. Introduction

Earthquakes (EQs) exhibit complex correlations in time, space and magnitude, e.g., [1,2,3,4,5,6,7,8,9,10]. The observed EQ scaling laws point to [11,12,13] the existence of phenomena closely associated with the proximity of the system to a critical point (the mainshock is the new phase [13,14]). In this frame, the order parameter $\kappa_{1}$ of seismicity is the quantity by means of which one can identify the approach of the dynamical system to the critical point. Such a parameter has been introduced [15] upon analyzing the seismicity, in a new time domain, termed natural time [13,14,16,17,18] (see below) which has found useful applications in diverse fields [13,19,20]. This analysis unveils hidden properties in time series of complex systems [13] and has recently been also used by Turcotte and coworkers as basis of a new methodology to estimate the current seismic risk level [21,22,23,24,25,26,27,28].

In a time series comprising *N* EQs, the natural time $\chi_{k}$ for the occurrence of the *k*-th EQ of energy $Q_{k}$ is defined as $\chi_{k}=k/N$, i.e., we ignore the time intervals between consecutive events, but preserve their order as well their energy $Q_{k}$. In natural time analysis, the evolution of the pair $\left(\chi_{k},p_{k}\right)$ is studied, where $p_{k}=Q_{k}/\sum_{n=1}^{N}Q_{n}$ is the normalized energy and $Q_{k}$ is estimated by means of the relation [29] $Q_{k}\propto 10^{1.5M_{k}}$, where $M_{k}$ stands for the EQ magnitude. It has been argued [15] that the variance $\kappa_{1}=\langle \chi^{2}\rangle -{\langle \chi \rangle }^{2}$ of natural time χ weighted for $p_{k}$, namely

(1) $$\kappa_{1}=\sum_{k=1}^{N}p_{k}{\left(\chi_{k}\right)}^{2}-{\left(\sum_{k=1}^{N}p_{k}\chi_{k}\right)}^{2},$$

may serve as an order parameter of seismicity. The entropy *S* in natural time is defined [18] by

(2) S≡〈χlnχ〉−〈χ〉ln〈χ〉

where the brackets $\langle \ldots \rangle \equiv \sum \left(\ldots \right)p_{k}$ denote averages with respect to the distribution $p_{k}$, i.e., $\langle f\left(\chi \right)\rangle \equiv \sum f\left(\chi_{k}\right)p_{k}$. Upon considering time reversal $\hat{T}$, i.e., $\hat{T}p_{k}=p_{N-k+1}$, the value *S* changes to a value $S_{-}$:

(3) $$\begin{array}{ccc} S_{-} & = & \sum_{k=1}^{N}p_{N-k+1}\left(\frac{k}{N}\right)\ln\left(\frac{k}{N}\right)\\ & - & \left(\sum_{k=1}^{N}\frac{k}{N}p_{N-k+1}\right)\ln\left[\sum_{l=1}^{N}\frac{l}{N}p_{N-l+1}\right] \end{array}$$

The physical meaning of the entropy change $\Delta S\equiv S-S_{-}$ in natural time under time reversal has been discussed in References [13,19].

The study of the fluctuations β of this order parameter of seismicity reveals challenging results. To compute $\kappa_{1}$ fluctuations, we follow the procedure described in detail in References [30,31] by using a sliding natural time window of constant length, i.e., consisting of a number *W* of EQs that would occur on average within the crucial scale [32] of a few months or so, which is the lead time of Seismic Electric Signals (SES) activities. These are series of low frequency transient changes of the electric field of the Earth [33,34] that are detected before major EQs (both in Japan [35] and Greece [36,37,38]). We then compute the average value $\mu_{W}\left(\kappa_{1}\right)$ and the standard deviation $\sigma_{W}\left(\kappa_{1}\right)$ of the ensemble of $\kappa_{1}$ obtained. The quantity

(4) $$\beta_{W}\equiv \sigma {}_{W}\left(\kappa_{1}\right)/\mu {}_{W}\left(\kappa_{1}\right)$$

is termed [13] variability of $\kappa_{1}$. The time evolution of the $\beta_{W}$ value can then be pursued by sliding the natural time window of *W* consecutive EQs, event by event, through the EQ catalog and assigning to its value the occurrence time of the EQ which follows the last EQ of the window in the EQ catalog. The corresponding minimum value is labeled $\beta_{W,min}$. Please note that $\beta_{W}$ of Equation (4) is reminiscent [39] of the square root of the Ginzburg criterion introduced within the frame of the mean field theories of the Ginzburg-Landau type, e.g., see p. 175 of Goldenfeld [40], discussed also by Holliday et al. [12].

**Figure 1:**
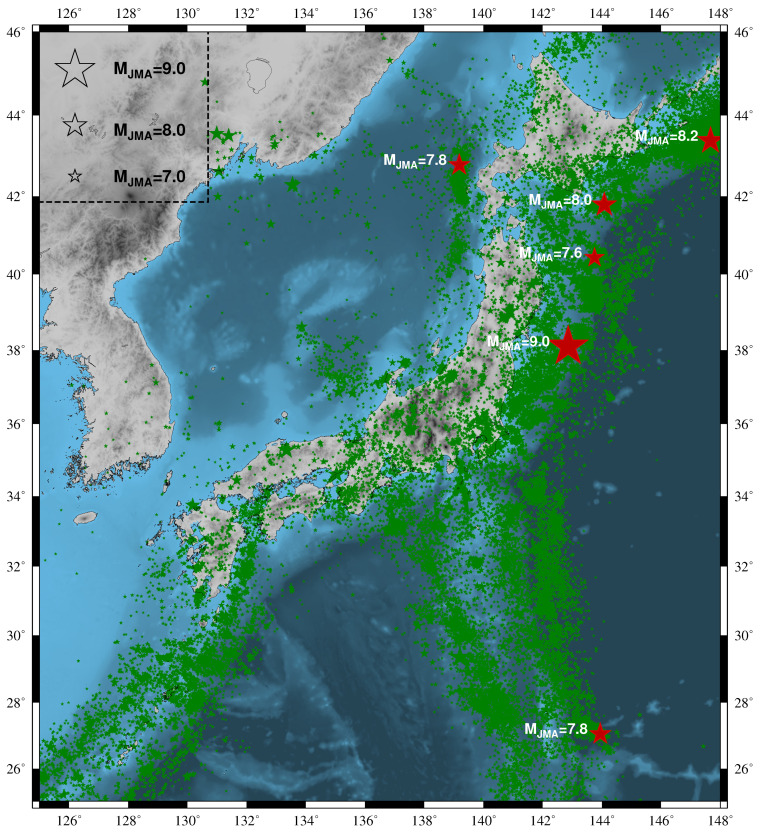
(color online) Epicenters (red stars) of all six shallow EQs with M ≥ 7.6 within the area N${}_{25}^{46}$ E${}_{125}^{148}$ since 1 January 1984 until the M9 Tohoku EQ. The smaller green stars indicate the epicenters of all M ≥ 3.5 EQs.

**Figure 2:**
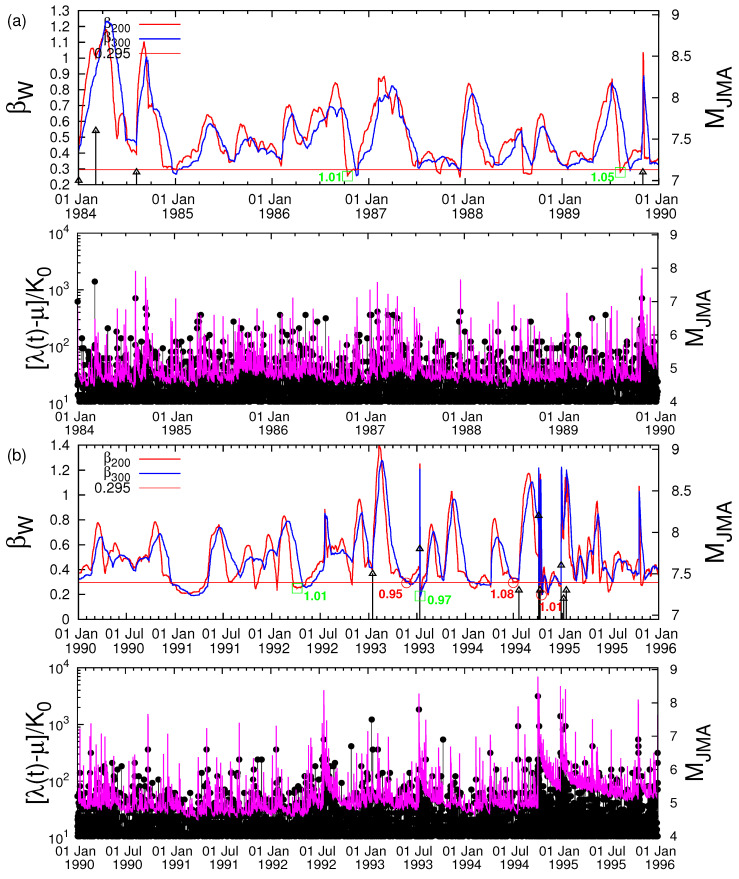

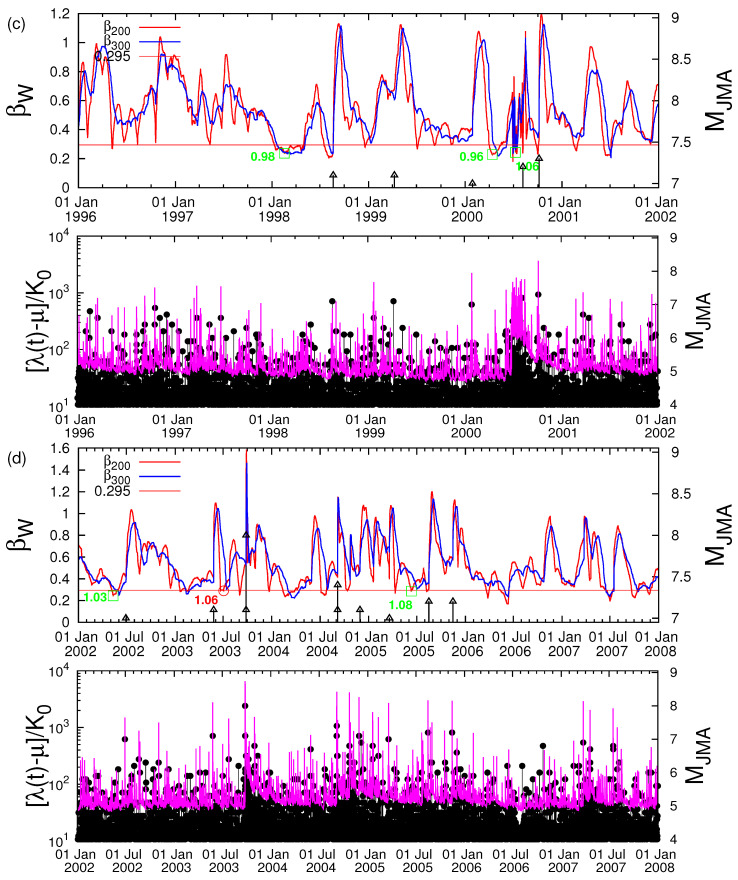

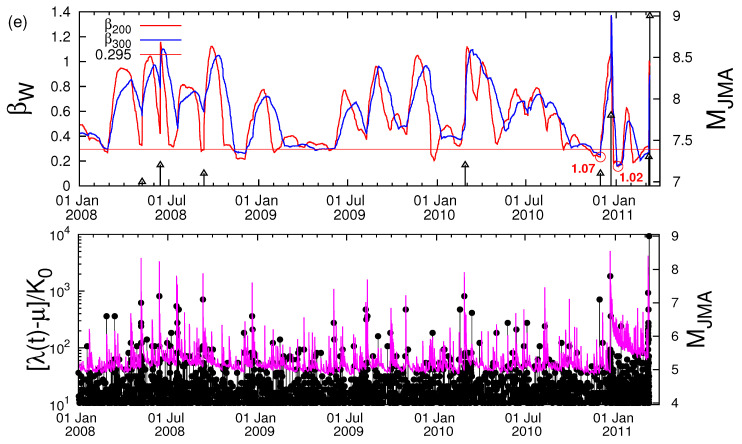
(color online) Variability β versus the conventional time depicted in consecutive 6 year periods in the upper graphs of the panels (**a**–**e**), respectively. The six minima preceded the M ≥ 7.6 EQs are marked by red circles at the $\beta_{200}$ curve while the nine minima followed by smaller EQs by green squares again at the $\beta_{200}$ curve; the values of $\beta_{300,min}/\beta_{200,min}$ are also written in red and green, respectively (see, e.g., Tables 1 and 2 of Reference [30]). No data are plotted in (e) after M9 Tohoku EQ. The horizontal red line corresponds to the shallowest $\beta_{200}$ minimum that preceded a M ≥ 7.6 EQ and the EQs are marked as black arrows whose magnitude can be read in the right scale. In addition, below the variability graph in each panel, we depict the time dependent seismicity rate $\left(\lambda \left(t\right)-\mu \right)/K_{0}=\sum_{t_{i}<t}\exp\left[\alpha (M_{i}-M_{c})\right]/{\left(t-t_{i}+c\right)}^{p}$ of the temporal epidemic-type aftershock sequence (ETAS) model [47,48,49] according to Equation (1) of Ogata et al. [50] together with the seismicity (black vertical lines ending at circles whose magnitude can be read in the right scale). The ETAS model parameters (α,p,c) are the same as those presented in Figure 2a in Reference [50].

The following results were revealed: Using the Japan Meteorological Agency (JMA) seismic catalog [30] and considering all EQs of magnitude $M_{\mathrm{JMA}}\geq 3.5$ from 1 January 1984 to 11 March 2011 (the time of the M9 Tohoku EQ) within the area 25${}^{{}^{\circ}}$–46${}^{{}^{\circ}}$ E, 125–148${}^{{}^{\circ}}$ E (Figure 1), fifteen distinct minima—observed simultaneously (see, e.g., Appendix A of Reference [31]) at $\beta_{200}$ and $\beta_{300}$ with a ratio $\beta_{300,min}/\beta_{200,min}$ in the range 0.95 to 1.08 and $\beta_{200,min}\leq 0.295$—of the fluctuations of the order parameter of seismicity were found 1 to around 3 months before large EQs. All shallow EQs of magnitude 7.6 or larger during this 27 year period (see Figure 1) were preceded by six (out of 15) of these minima $\beta_{W,min}$ (see Figure 2), the spatiotemporal variations of which also reveal an estimate of the candidate epicentral area [41]. Remarkably, among the minima, the deepest minimum was observed around 5 January 2011 (which is almost two weeks after the minimization on 22 December 2010 of the entropy change under time reversal [42]), i.e., around two months before the M9 Tohoku EQ.

Using Monte Carlo calculations, Sarlis et al. [43] showed that the probability to achieve the above results by chance is of the order of $10^{-5}$ (we shall return to this point later). This conclusion was also recently strengthened by Christopoulos et al. [44] using the event coincidence analysis (ECA) [45]. It is the scope of the present paper to investigate the diagnostic accuracy of the precursory minima $\beta_{W,min}$ before major EQs in Japan during 1984–2011 by employing the area under the receiver operating characteristic (ROC) [46] curve (AUC), which is a very recent technique for judging the quality of binary predictions.

## 2. Receiver Operating Characteristics Technique

**Figure 3:**
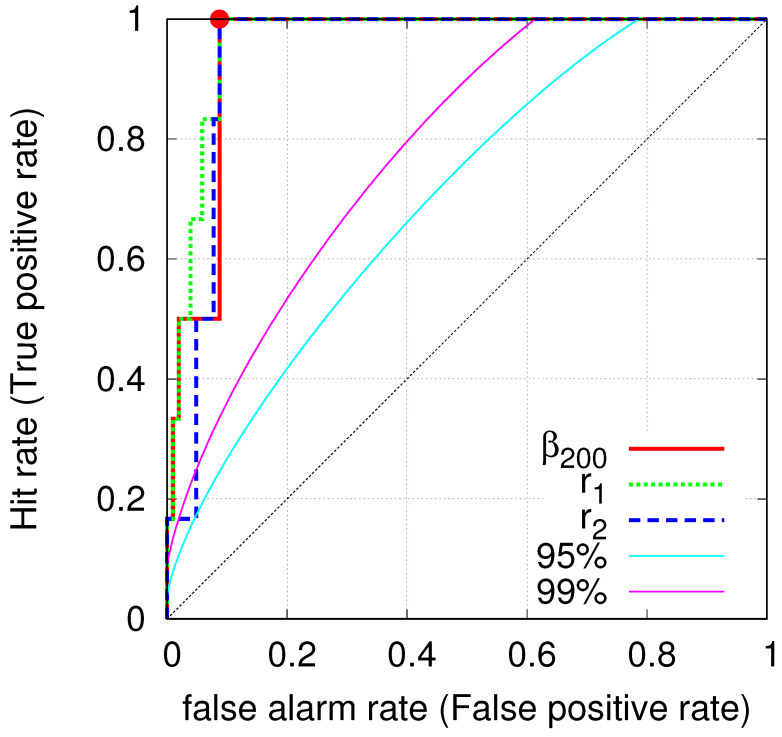
ROC curves resulting from the study of the minima of the fluctuations of the order parameter of seismicity in Japan. For all the three ROCs, the corresponding AUCs have a mean value 0.95 reflecting an outstanding discrimination (see p. 162 of Reference [53]). The cyan and magenta lines are the *k*-ellipses that correspond [55] to the 95% and 99% confidence intervals. They indicate how far away from the diagonal the ROC curve of a random predictor may scatter with probability 1/20 or 1/100.

The performance of a diagnostic test in the case of a binary predictor can be evaluated using the measures of sensitivity [51] (where sensitivity = (True positives)/(True positives + False negatives)) and specificity (where specificity = (True negatives)/(True negatives + False positives)) (e.g., Reference [52]). This is achieved by a ROC curve that includes all the possible decision thresholds from a diagnostic test results. Simply defined, a ROC curve is a plot of the sensitivity versus the quantity ‘1-specificity’, i.e., the False positive rate=(False positives)/(True negatives + False positives), of a diagnostic test. Hence, a ROC diagram depicts the sensitivity or hit rate (or True positive rate) versus the False positive rate (or false alarm rate) thus showing the trade-off between hits and false alarms [46]. The AUC is an effective way to summarize the overall diagnostic accuracy of the test. It takes a value of 1 for a perfectly accurate test. An AUC of 0.5 suggests no discrimination (i.e., ROC curve falls on the diagonal), 0.7 to 0.8 is considered acceptable, 0.8 to 0.9 is considered excellent, and more than 0.9 is considered outstanding [51]; see also Chapter 5, pp. 160–164 of Reference [53]. Moreover, as shown by Mason and Graham [54] AUC can be used to estimate the statistical significance of the prediction scheme. Recently, a method was proposed [55] that can estimate the AUC—and hence the statistical significance—corresponding to an operating point in the ROC plane using the so-called *k*-ellipses which are constructed on the basis of confidence ellipses. According to this method, when only one operating point of a prediction method is known, then the AUC of the corresponding ROC curve can be approximated by the AUC of the *k*-ellipse that passes through this point, e.g., see Figure 3 in Reference [55].

By means of the latter method, the AUC corresponding to the precursory variations of the Earth’s electric and magnetic field that precede EQs in Greece in the 1980s, see, e.g., Dologlou [56], (i.e., the two points depicted in Figure 4 of Reference [57]) and EQs in Japan in the 2000s, see, e.g., Han et al. [58], (i.e., the two points in Figure 8a,b of Reference [57]) can be estimated. They are 0.625 and 0.658 for the two operating points in Figure 4 of Reference [57] and 0.869 and 0.943 for the the two operating points shown in Figure 8a,b of Reference [57], respectively. These four AUC exceed the one shown in Figure 11 of Reference [59] which has been calculated for the relationship between magnetic field pulses and EQs in California.

## 3. Data Analyzed

Here, as in References [30,41], the JMA seismic catalog was used and the energy $Q_{k}$ of each EQ was obtained from $M_{\mathrm{JMA}}$ after converting [60] to the moment magnitude $M_{W}$ defined by Hanks and Kanamori [61]. We consider all EQs—by setting a threshold $M_{\mathrm{JMA}}$ = 3.5 in order to assure data completeness (see Reference [30]; see also Figure 6 in Nanjo et al. [62])—from 1 January 1984 to the M9 Tohoku EQ occurrence on 11 March 2011, within the area N${}_{25}^{46}$ E${}_{125}^{148}$, which covers the whole Japanese region (see Figure 1). Since 47,204 EQs occurred in this period of about 326 months, we have on average $\approx 10^{2}$ EQs per month, thus we choose the natural time window lengths *W* = 200 and 300 for the calculation of $\beta_{W}$ that would correspond on average to a few months period in accordance with the SES activities observations, as already mentioned.

## 4. Results

In Figure 2, we plot the variability $\beta_{W}$ for *W*=200 (red) and *W*=300 (blue) (left scale) along with all $M_{\mathrm{JMA}}\geq 7.0$ ($M_{\mathrm{JMA}}$ in the right scale) versus the conventional time in four consecutive 6 year periods (a), (b), (c), (d), respectively, and one period (e) of almost 3 years. The six minima $\beta_{W,min}$ preceeding the $M_{\mathrm{JMA}}\geq 7.6$ EQs (see Table 1 of Reference [30]) are marked with red circles, while the nine followed by the $M_{\mathrm{JMA}}\geq 6.4$ EQs (see Table 2 of Reference [30]) with green squares. Other minima below the threshold (i.e., $\beta_{200,min}\leq 0.295$) that have not been marked by red circles or green squares, did not obey any of the following criteria (selected for reasons explained in detail by Sarlis et al. [30] and Varotsos et al. [31]): $\beta_{200}$ and $\beta_{300}$ should appear simultaneously (see, e.g., Appendix A of Reference [31]) with a ratio $\beta_{300,min}/\beta_{200,min}$ in the range 0.95 to 1.08.

Figure 3 depicts the ROC graph, i.e., the plot of True positive rate versus the False positive rate, as a function of the total rate of alarms with an alarm period of 3 months, which is tuned by a threshold in the predictor. In particular, we vary the value of the shallowest $\beta_{200,min}$ from 0.295 down to 0.157 and we obtain the ROC depicted by the red solid line which has AUC=0.951. Similarly, upon increasing the value of the lower threshold $r_{1}$ of the ratio $\beta_{300,min}$/$\beta_{200,min}$ from 0.95 to 1.08, we obtain the green dotted ROC with AUC = 0.965. Finally, the dashed blue ROC with AUC = 0.943 corresponds to the case that we decrease the maximum value of the ratio $\beta_{300,min}$/$\beta_{200,min}$ from 1.08 down to 0.95. In the same figure, we also depict the *k*-ellipses that correspond [55] to the 95% and 99% confidence intervals. We observe that in all these three cases the observed ROCs exceed the 99% confidence interval exhibiting statistical significance (see also below).

## 5. Discussion

AUC is currently considered [51] an effective way to summarize the overall accuracy of a diagnostic test, as already mentioned in Section 2. It is characterized outstanding when AUC is more than 0.9 [51], which is of course the present case of $\beta_{W,min}$ since the AUC computed here is around 0.95. This result corroborates with the fact that the statistical significance of the precursory nature of $\beta_{W,min}$ has been shown by two other independent procedures, in particular Monte Carlo calculations in Reference [43] and ECA in Reference [44].

The above result (AUC ≈ 0.95) can be also used to to estimate the overall diagnostic accuracy of SES and the associated Earth’s magnetic field variations, in view of the following experimental fact emerged from measurements of independent research groups: Observations show that there exists simultaneous appearance of the minima of the fluctuations of the order parameter of seismicity with the initiation of SES activities, before major EQs in Japan and Greece. This was [63] the first time in the literature that before major EQs anomalous changes are found to appear simultaneously (as well as they are also linked in space) in two independent data sets of different geophysical observables (geoelectrical measurements and seismicity). Focusing on measurements in Japan for example, let us consider the volcanic seismic swarm activity in 2000 in the Izu Island region, which was then characterized by JMA as being the largest EQ swarm ever recorded [64], and the M9 Tohoku EQ on 11 March 2011 which is the strongest EQ ever recorded in Japan. Specifically, a straightforward analysis of the JMA seismic catalog in natural time, by employing a sliding natural time window comprising the number of events that would occur in a few months, it was observed [63] that the fluctuations of the order parameter of seismicity exhibit a clearly detectable minimum at the time of the initiation of the pronounced SES activity identified by Uyeda et al. [35,65] almost two months before the onset of the volcanic-seismic swarm activity in 2000 in the Izu Island region, Japan. Concerning the $\beta_{W,min}$ before the M9 Tohoku EQ, this was observed [30] on 5 January 2011 being the deepest minimum $\beta_{W,min}$ during the period from 1 January 1984 until the M9 Tohoku EQ occurrence, as mentioned. This date almost coincides with the detection of anomalous magnetic field variations on the z component during the period from 4 to 14 January 2011 at two measuring sites lying at epicentral distances of around 130 km [66,67,68] pointing to the initiation of an SES activity (as observed for SES activities in Greece, see, e.g., pp. 8–9 of Reference [37], in which the associated magnetic field variations are clearly detectable at epicentral distances up to around 150–200 km for EQs of magnitude 6.0 or larger). This occurred almost two weeks after the observation [42] on 22 December 2010 of the minimum $\Delta S_{min}$ of the entropy change ΔS of seismicity under time reversal.

The probability to obtain such a minimum by chance was shown to be approximately 3%, thus it is statistically significant. Such a minimum is of precursory nature signaling that a large EQ is impending according to the conclusions deduced from the natural time analysis of the Olami-Feder-Christensen (OFC) model for EQs [69], which is probably [70] the most studied non-conservative self-organized criticality (SOC) model. In particular, it has been shown that ΔS exhibits a clear minimum [13] (or maximum if we define [71] $\Delta S\equiv S_{-}-S$ instead of $\Delta S\equiv S-S_{-}$) before a large avalanche in the OFC model, which corresponds to a large EQ. The minimum $\Delta S_{min}$ observed on 22 December 2010 has been recently shown to be of profound importance in identifying the occurrence time of the M9 Tohoku EQ [72,73]. It was also found [74] (see p. 321) that it corresponds to the first stage of the physical model for the SES generation according to which an excess stress disturbance starts gradually increasing until reaching the critical stress. This minimum was accompanied by an abrupt increase of the order parameter of seismicity [75] to which we now turn in view of the interesting property that it exhibits a natural time scale dependence that has a functional form suggested by Penrose and coworkers [76] consistent with the phase transition kinetics of Lifshitz and Slyozov [77]. Beyond the six (out of 15) $\beta_{W}$ minima preceding all shallow EQs of magnitude 7.6 or larger, an inspection of Figure 2 reveals that a $\beta_{W}$ increase appears upon the occurrence of a major EQ [75]. In particular, a careful study of Figure 2 shows that there exist six prominent fluctuations increases of $\beta_{W}$ (higher than 1.2) before the M9 Tohoku EQ. These are accompanied by major EQs and specifically the $\beta_{W}$ increase observed in the beginning of 1993 after the M7.5 EQ on 15 January 1993 at 42.92${}^{{}^{\circ}}$ N 144.35${}^{{}^{\circ}}$ E and the increases of $\beta_{W}$ associated with all shallow EQs of magnitude 7.6 or larger that occurred from 1 January 1984 until the M9 Tohoku EQ occurrence, which are the following: The M7.8 EQ on 12 July 1993, the M8.2 EQ on 4 October 1994, the M7.6 EQ on 28 December 1994, the M8.0 EQ in 2003, and the M7.8 EQ in 2010. The two larger are in 2003 and in 2010: First, the large increase of $\beta_{W}$ in 2003 appears upon the occurrence of the M8 EQ on 26 September 2003 (attaining its maximum on 28 September 2003, see Figure 2b of Reference [75]). Second, the increase of $\beta_{W}$ on 22 December 2010 is observed upon the occurrence on the same day of the M7.8 near Chichi-jima EQ. The latter, i.e., the one in 2010, appears almost simultaneously with the minimum $\Delta S_{min}$ the entropy change of seismicity under time reversal, which occurs also on 22 December 2010 as it was found upon applying the procedure of Reference [42] to the entire Japanese region seismic data.

In order to assure the simultaneity of the minima $\beta_{W,min}$ with the initiation of SES activities as well as their statistical significance, attention is drawn to a few misunderstandings explained below:

First, only the minima $\beta_{W,min}$ of the order parameter fluctuations defined in Equation (4), but not the minima of $\kappa_{1}$ (as claimed in Reference [78]), are of precursory nature [30,43,44].

Second, as mentioned in the introduction, the probability to achieve the results concerning $\beta_{W,min}$ of Reference [30] by chance is of the order $10^{-5}$. This value was obtained both by Monte Carlo calculations as well as with the ROC technique. For example, in the latter technique, the calculation was made as follows: Sarlis et al. [30] found 15 distinct minima $\beta_{W,min}$ during the 27 year period from 1 January 1984 to 11 March 2011 and six of these minima preceded (by 1–3 months) all shallow EQs of magnitude 7.6 or larger. In general, the ROC curves exhibit fluctuations which depend on the positive P cases (the number of significant events, i.e., the 6 EQs of magnitude 7.6 or larger in the present case) and the negative Q cases (the number of non-significant events, i.e., M < 7.6) to be predicted. Thus, by dividing the 27 year period by almost 3 months, we have 109 three-month periods (i.e., P + Q = 109) out of which only six included significant events (P = 6). Hence, all six significant events were successfully predicted, thus the hit rate is 100%. The  nine minima that were followed within three months by smaller EQs may be considered false alarms, thus the false alarm rate (False positive rate) is 9/103 ≈ 8.74%. A straightforward calculation by using the FORTRAN code VISROC.f, provided in Reference [55], gives that the probability *p* to obtain this operation point (8.74%, 100%) by chance based on k-ellipses is of the order of $10^{-5}$. In other words, this result ($p=10^{-5}$) demonstrates that it is incorrect to claim [78] that since there exists an abundance of false positives (i.e., 6 true positives while 9 false positives in the present case) the method of identifying $\beta_{W,min}$ that are simultaneous with SES activities is unusable without, however, paying attention to its statistical significance (cf. This can be also shown analytically by applying the formulation given in the Appendix of Reference [79]). Such a line of thinking (abundance of false positives) is in contrast with the proper use of ROC curves as discussed in detail in Section 6 of Reference [46], see Equation (1) there, according to which the selection of the operating point should be selected on the basis of the trade of the losses in case of false negative and the cost of a false positive.

Third, the aforementioned nine false alarms (false positives) could have been avoided by following the procedure developed in Reference [31] in which the analysis of seismicity of Japan and the computation of $\beta_{W}$ was made in two different areas N${}_{25^{{}^{\circ}}}^{46^{{}^{\circ}}}$E${}_{125^{{}^{\circ}}}^{146^{{}^{\circ}}}$ and N${}_{25^{{}^{\circ}}}^{46^{{}^{\circ}}}$E${}_{125^{{}^{\circ}}}^{148^{{}^{\circ}}}$ (instead of the single area N${}_{25^{{}^{\circ}}}^{46^{{}^{\circ}}}$E${}_{125^{{}^{\circ}}}^{148^{{}^{\circ}}}$ in Reference [30]) which are compatible with the results obtained in Reference [8] by means of EQ networks based on similar activity patterns. Comparing the results between these two areas all false alarms have been excluded since they did not simultaneously obey the criteria applied to both areas. This way, as true positives were selected only the minima $\beta_{W,min}$ preceding the strongest EQs in the smaller area, while the remaining minima preceding EQs of smaller magnitude were excluded.

Fourth, concerning the detection of true SES activities, we clarify that electrical disturbances that may look to be similar to SES activities, but not obeying the criteria developed in References [17,18,36] to distinguish them from noise, are not classified as SES activities.

## 6. Conclusions

Upon analysing the seismicity of Japan during an almost 27 year period, i.e., from 1 January 1984 to 11 March 2011 (the time of the M9 Tohoku EQ occurrence), we identify the minima $\beta_{W,min}$ of the fluctuations of the order parameter of seismicity (which are in general accompanied by simultaneous SES activities). Focusing on the area under the receiver operating characteristic curve, which is currently considered an effective way to summarize the overall diagnostic accuracy of a test, we found a value close to AUC = 0.95. This shows that the precursory nature of these minima is outstanding, after taking into account that AUC takes the value of 1 for a perfectly accurate diagnostic test.

## Abbreviations

The following abbreviations are used in this manuscript.

AUC	Area under the ROC curve
ECA	Event coincidence analysis
EQ	Earthquake
JMA	Japan Meteorological Agency
ROC	Receiver operating characteristic
SES	Seismic electric signals

## References

[1] Telesca L., Lapenna V., Macchiato M. (2003). Spatial variability of the time-correlated behaviour in Italian seismicity. Earth Planet. Sci. Lett..

[2] Huang Q. (2008). Seismicity changes prior to the Ms8.0 Wenchuan earthquake in Sichuan, China. Geophys. Res. Lett..

[3] Lennartz S., Livina V.N., Bunde A., Havlin S. (2008). Long-term memory in earthquakes and the distribution of interoccurrence times. EPL.

[4] Telesca L. (2010). Analysis of Italian seismicity by using a non-extensive approach. Tectonophysics.

[5] Lennartz S., Bunde A., Turcotte D.L. (2011). Modelling seismic catalogues by cascade models: Do we need long-term magnitude correlations?. Geophys. J. Int..

[6] Telesca L., Lovallo M., Amin Mohamed A.E.E., ElGabry M., El-hady S., Abou Elenean K.M., ElShafey Fat ElBary R. (2012). Investigating the time-scaling behavior of the 2004–2010 seismicity of Aswan area (Egypt) by means of the Allan factor statistics and the detrended fluctuation analysis. Nat. Hazards Earth Syst. Sci..

[7] Tiampo K.F., Shcherbakov R. (2012). Seismicity-based earthquake forecasting techniques: Ten years of progress. Tectonophysics.

[8] Tenenbaum J.N., Havlin S., Stanley H.E. (2012). Earthquake networks based on similar activity patterns. Phys. Rev. E.

[9] Vallianatos F., Michas G., Papadakis G. (2014). Non-extensive and natural time analysis of seismicity before the Mw6.4, October 12, 2013 earthquake in the South West segment of the Hellenic Arc. Physica A.

[10] Sarlis N.V., Skordas E.S., Mintzelas A., Papadopoulou K.A. (2018). Micro-scale, mid-scale, and macro-scale in global seismicity identified by empirical mode decomposition and their multifractal characteristics. Sci. Rep..

[11] Carlson J.M., Langer J.S., Shaw B.E. (1994). Dynamics of earthquake faults. Rev. Mod. Phys..

[12] Holliday J.R., Rundle J.B., Turcotte D.L., Klein W., Tiampo K.F., Donnellan A. (2006). Space-Time Clustering and Correlations of Major Earthquakes. Phys. Rev. Lett..

[13] Varotsos P.A., Sarlis N.V., Skordas E.S. (2011). Natural Time Analysis: The New View of Time. Precursory Seismic Electric Signals, Earthquakes and other Complex Time-Series.

[14] Varotsos P.A., Sarlis N.V., Skordas E.S. (2001). Spatio-Temporal complexity aspects on the interrelation between Seismic Electric Signals and Seismicity. Pract. Athens Acad..

[15] Varotsos P.A., Sarlis N.V., Tanaka H.K., Skordas E.S. (2005). Similarity of fluctuations in correlated systems: The case of seismicity. Phys. Rev. E.

[16] Varotsos P.A., Sarlis N.V., Skordas E.S. (2002). Long-range correlations in the electric signals that precede rupture. Phys. Rev. E.

[17] Varotsos P.A., Sarlis N.V., Skordas E.S. (2003). Long-range correlations in the electric signals the precede rupture: Further investigations. Phys. Rev. E.

[18] Varotsos P.A., Sarlis N.V., Skordas E.S. (2003). Attempt to distinguish electric signals of a dichotomous nature. Phys. Rev. E.

[19] Varotsos P.A., Sarlis N.V., Skordas E.S., Lazaridou M.S. (2007). Identifying sudden cardiac death risk and specifying its occurrence time by analyzing electrocardiograms in natural time. Appl. Phys. Lett..

[20] Baldoumas G., Peschos D., Tatsis G., Chronopoulos S.K., Christofilakis V., Kostarakis P., Varotsos P., Sarlis N.V., Skordas E.S., Bechlioulis A. (2019). A Prototype Photoplethysmography Electronic Device that Distinguishes Congestive Heart Failure from Healthy Individuals by Applying Natural Time Analysis. Electronics.

[21] Rundle J.B., Turcotte D.L., Donnellan A., Grant Ludwig L., Luginbuhl M., Gong G. (2016). Nowcasting earthquakes. Earth Space Sci..

[22] Rundle J.B., Luginbuhl M., Giguere A., Turcotte D.L. (2018). Natural Time, Nowcasting and the Physics of Earthquakes: Estimation of Seismic Risk to Global Megacities. Pure Appl. Geophys..

[23] Luginbuhl M., Rundle J.B., Hawkins A., Turcotte D.L. (2018). Nowcasting Earthquakes: A Comparison of Induced Earthquakes in Oklahoma and at the Geysers, California. Pure Appl. Geophys..

[24] Luginbuhl M., Rundle J.B., Turcotte D.L. (2018). Natural Time and Nowcasting Earthquakes: Are Large Global Earthquakes Temporally Clustered?. Pure Appl. Geophys..

[25] Luginbuhl M., Rundle J.B., Turcotte D.L. (2018). Statistical physics models for aftershocks and induced seismicity. Phil. Trans. R. Soc. A.

[26] Luginbuhl M., Rundle J.B., Turcotte D.L. (2018). Natural time and nowcasting induced seismicity at the Groningen gas field in the Netherlands. Geophys. J. Int..

[27] Rundle J.B., Giguere A., Turcotte D.L., Crutchfield J.P., Donnellan A. (2019). Global Seismic Nowcasting With Shannon Information Entropy. Earth Space Sci..

[28] Rundle J.B., Luginbuhl M., Khapikova P., Turcotte D.L., Donnellan A., McKim G. (2019). Nowcasting Great Global Earthquake and Tsunami Sources. Pure Appl. Geophys..

[29] Kanamori H. (1978). Quantification of Earthquakes. Nature.

[30] Sarlis N.V., Skordas E.S., Varotsos P.A., Nagao T., Kamogawa M., Tanaka H., Uyeda S. (2013). Minimum of the order parameter fluctuations of seismicity before major earthquakes in Japan. Proc. Natl. Acad. Sci. USA.

[31] Varotsos P.A., Sarlis N.V., Skordas E.S. (2014). Study of the temporal correlations in the magnitude time series before major earthquakes in Japan. J. Geophys. Res..

[32] Varotsos P., Sarlis N., Skordas E. (2011). Scale-specific order parameter fluctuations of seismicity in natural time before mainshocks. EPL.

[33] Varotsos P., Alexopoulos K. (1984). Physical Properties of the variations of the electric field of the Earth preceding earthquakes, I. Tectonophysics.

[34] Varotsos P., Alexopoulos K., Nomicos K., Lazaridou M. (1986). Earthquake prediction and electric signals. Nature.

[35] Uyeda S., Kamogawa M., Tanaka H. (2009). Analysis of electrical activity and seismicity in the natural time domain for the volcanic-seismic swarm activity in 2000 in the Izu Island region, Japan. J. Geophys. Res..

[36] Varotsos P., Lazaridou M. (1991). Latest aspects of earthquake prediction in Greece based on Seismic Electric Signals. Tectonophysics.

[37] Varotsos P., Alexopoulos K., Lazaridou M. (1993). Latest aspects of earthquake prediction in Greece based on Seismic Electric Signals, II. Tectonophysics.

[38] Sarlis N.V., Skordas E.S., Lazaridou M.S., Varotsos P.A. (2008). Investigation of seismicity after the initiation of a Seismic Electric Signal activity until the main shock. Proc. Jpn. Acad. Ser. B Phys. Biol. Sci..

[39] Mintzelas A., Sarlis N. (2019). Minima of the fluctuations of the order parameter of seismicity and earthquake networks based on similar activity patterns. Physica A.

[40] Goldenfeld N. (2018). Lectures on Phase Transitions and the Renormalization Group.

[41] Sarlis N.V., Skordas E.S., Varotsos P.A., Nagao T., Kamogawa M., Uyeda S. (2015). Spatiotemporal variations of seismicity before major earthquakes in the Japanese area and their relation with the epicentral locations. Proc. Natl. Acad. Sci. USA.

[42] Sarlis N.V., Skordas E.S., Varotsos P.A. (2018). A remarkable change of the entropy of seismicity in natural time under time reversal before the super-giant M9 Tohoku earthquake on 11 March 2011. EPL.

[43] Sarlis N.V., Skordas E.S., Christopoulos S.R.G., Varotsos P.A. (2016). Statistical Significance of Minimum of the Order Parameter Fluctuations of Seismicity Before Major Earthquakes in Japan. Pure Appl. Geophys..

[44] Christopoulos S.R.G., Skordas E.S., Sarlis N.V. (2020). On the Statistical Significance of the Variability Minima of the Order Parameter of Seismicity by Means of Event Coincidence Analysis. Appl. Sci..

[45] Donges J., Schleussner C.F., Siegmund J., Donner R. (2016). Event coincidence analysis for quantifying statistical interrelationships between event time series. Eur. Phys. J. Spec. Top..

[46] Fawcett T. (2006). An introduction to ROC analysis. Pattern Recogn. Lett..

[47] Ogata Y. (1985). Statistical Models for Earthquake Occurrences and Residual Analysis for Point Processes. Research Memorandum (Technical Report). https://www.ism.ac.jp/editsec/resmemo-j.html.

[48] Ogata Y. (1988). Statistical Models for Earthquake Occurrences and Residual Analysis for Point Processes. J. Am. Statist. Assoc..

[49] Ogata Y. (1989). Statistical model for standard seismicity and detection of anomalies by residual analysis. Tectonophysics.

[50] Ogata Y., Katsura K., Tsuruoka H., Hirata N. (2019). High-resolution 3D earthquake forecasting beneath the greater Tokyo area. Earth Planets Space.

[51] Mandrekar J.N. (2010). Receiver Operating Characteristic Curve in Diagnostic Test Assessment. J. Thorac. Oncol..

[52] Lalkhen A.G., McCluskey A. (2008). Clinical tests: Sensitivity and specificity. CEACCP.

[53] Hosmer D.W., Lemeshow S. (2000). Applied Logistic Regression.

[54] Mason S.J., Graham N.E. (2002). Areas beneath the relative operating characteristics (ROC) and relative operating levels (ROL) curves: Statistical significance and interpretation. Quart. J. R. Meteor. Soc..

[55] Sarlis N.V., Christopoulos S.R.G. (2014). Visualization of the significance of Receiver Operating Characteristics based on confidence ellipses. Comput. Phys. Commun..

[56] Dologlou E. (1993). A three year continuous sample of officially documented predictions issued in Greece using the VAN method: 1987–1989. Tectonophysics.

[57] Sarlis N.V. (2018). Statistical Significance of Earth’s Electric and Magnetic Field Variations Preceding Earthquakes in Greece and Japan Revisited. Entropy.

[58] Han P., Hattori K., Zhuang J., Chen C.H., Liu J.Y., Yoshida S. (2017). Evaluation of ULF seismo-magnetic phenomena in Kakioka, Japan by using Molchan’s error diagram. Geophys. J. Int..

[59] Kappler K., Schneider D., MacLean L., Bleier T., Lemon J. (2019). An algorithmic framework for investigating the temporal relationship of magnetic field pulses and earthquakes applied to California. Comput. Geosci..

[60] Tanaka H.K., Varotsos P.A., Sarlis N.V., Skordas E.S. (2004). A plausible universal behaviour of earthquakes in the natural time-domain. Proc. Jpn. Acad. Ser. B Phys. Biol. Sci..

[61] Hanks T.C., Kanamori H. (1979). A moment magnitude scale. J. Geophys. Res..

[62] Nanjo K.Z., Ishibe T., Tsuruoka H., Schorlemmer D., Ishigaki Y., Hirata N. (2010). Analysis of the Completeness Magnitude and Seismic Network Coverage of Japan. Seismol. Soc. Am. Bull..

[63] Varotsos P.A., Sarlis N.V., Skordas E.S., Lazaridou M.S. (2013). Seismic Electric Signals: An additional fact showing their physical interconnection with seismicity. Tectonophysics.

[64] Japan Meteorological Agency (2000). Recent seismic activity in the Miyakejima and Niijima-Kozushima region, Japan -the largest earthquake swarm ever recorded-. Earth Planets Space.

[65] Uyeda S., Hayakawa M., Nagao T., Molchanov O., Hattori K., Orihara Y., Gotoh K., Akinaga Y., Tanaka H. (2002). Electric and magnetic phenomena observed before the volcano-seismic activity in 2000 in the Izu Island Region, Japan. Proc. Natl. Acad. Sci. USA.

[66] Xu G., Han P., Huang Q., Hattori K., Febriani F., Yamaguchi H. (2013). Anomalous behaviors of geomagnetic diurnal variations prior to the 2011 off the Pacific coast of Tohoku earthquake (Mw9.0). J. Asian Earth Sci..

[67] Han P., Hattori K., Xu G., Ashida R., Chen C.H., Febriani F., Yamaguchi H. (2015). Further investigations of geomagnetic diurnal variations associated with the 2011 off the Pacific coast of Tohoku earthquake (Mw 9.0). J. Asian Earth Sci..

[68] Han P., Hattori K., Huang Q., Hirooka S., Yoshino C. (2016). Spatiotemporal characteristics of the geomagnetic diurnal variation anomalies prior to the 2011 Tohoku earthquake (Mw 9.0) and the possible coupling of multiple pre-earthquake phenomena. J. Asian Earth Sci..

[69] Olami Z., Feder H.J.S., Christensen K. (1992). Self-organized criticality in a continuous, nonconservative cellular automaton modeling earthquakes. Phys. Rev. Lett..

[70] Ramos O., Altshuler E., Måløy K.J. (2006). Quasiperiodic Events in an Earthquake Model. Phys. Rev. Lett..

[71] Sarlis N., Skordas E., Varotsos P. (2011). The change of the entropy in natural time under time-reversal in the Olami-Feder-Christensen earthquake model. Tectonophysics.

[72] Skordas E.S., Sarlis N.V., Varotsos P.A. (2019). Identifying the occurrence time of an impending major earthquake by means of the fluctuations of the entropy change under time reversal. EPL.

[73] Varotsos P.A., Skordas E.S., Sarlis N.V. (2020). Fluctuations of the entropy change under time reversal: Further investigations on identifying the occurrence time of an impending major earthquake. EPL.

[74] Varotsos P.A., Sarlis N.V., Skordas E.S. (2019). Phenomena preceding major earthquakes interconnected through a physical model. Ann. Geophys..

[75] Varotsos P.A., Sarlis N.V., Skordas E.S. (2019). Natural time analysis: Important changes of the order parameter of seismicity preceding the 2011 M9 Tohoku earthquake in Japan. EPL.

[76] Penrose O., Lebowitz J.L., Marro J., Kalos M.H., Sur A. (1978). Growth of clusters in a first-order phase transition. J. Stat. Phys..

[77] Lifshitz I., Slyozov V. (1961). The kinetics of precipitation from supersaturated solid solutions. J. Phys. Chem. Solids.

[78] Helman D.S. (2020). Seismic electric signals (SES) and earthquakes: A review of an updated VAN method and competing hypotheses for SES generation and earthquake triggering. Phys. Earth Planet. Int..

[79] Varotsos P., Sarlis N., Lazaridou M. (1996). Reply to “Rebuttal to Reply by Varotsos and Lazaridou: Towards plainly successful prediction,” by Paul W. Burton. Geophys. Res. Lett..

